# Factors associated with regular physical exercise and consumption of fruits and vegetables among Mexican older adults

**DOI:** 10.1186/s12889-016-3628-2

**Published:** 2016-09-09

**Authors:** Svetlana V. Doubova, Sergio Sánchez-García, Claudia Infante-Castañeda, Ricardo Pérez-Cuevas

**Affiliations:** 1 Epidemiology and Health Services Research Unit, CMN Siglo XXI, Mexican Institute of Social Security, Av. Cuauhtemoc 330, Col. Doctores, Del. Cuauhtemoc, CP 06720 Mexico City, Mexico; 2 Epidemiology and Health Services Research Unit, Aging Area. CMN Siglo XXI, Mexican Social Security Institute, Av. Cuauhtemoc 330, Edificio CORSE 3er piso. Col. Doctores. Del. Cuauhtemoc, CP 06720 Mexico City, Mexico; 3 Instituto de Investigaciones Sociales. Universidad Nacional Autónoma de México, México City, Mexico; 4 Division of Social Protection and Health, Inter-American Development Bank, Mexico City, Mexico

**Keywords:** Older adults, Healthy behaviors, Fruit and vegetables consumption, Physical exercise, Mexico

## Abstract

**Background:**

To analyze the factors associated with regular physical exercise and routine consumption of fruits and vegetables, and both healthy behaviors among Mexican older adults.

**Methods:**

We conducted a secondary data analysis of the baseline data (2014) of the Study on Obesity, Sarcopenia and Fragility in older adults affiliated with the Mexican Institute of Social Security. The study included 948 adults who were ≥60 years of age. Multiple Poisson regression was performed.

**Results:**

Routine consumption of fruits and vegetables was reported by 53.8 % of older adults, 42.7 % reported engaging in regular physical exercise and 23.1 % reported participating in both types of healthy behaviors. Women, adults with a stable income, those with a self-perception of good health and those with a history of physical exercise at the age of 50 years had an increased likelihood of engaging in healthy eating and regular physical activity.

**Conclusions:**

Many older adults do not routinely consume fruits and vegetables or engage in regular physical exercise despite the fact that most have a fixed income and a social network. It is relevant to conduct research-based interventions that take into account the contextual factors to promote healthy behaviors.

## Background

The proportion of older adults in comparison with other age groups continues to increase worldwide. This increase is rapid in Latin America and Caribbean (LAC) countries [1]. Between 1950 and 2010, the proportion of persons aged 60+ years increased from 5.6 to 9.9 %. By 2100, this age group will account for >35 % of the population of the region [2]. Mexico follows the same tendency. Between 2010 and 2050, the proportion of older adults will increase from 5.2 to 21.2 % [3].

Additional years of life are often accompanied by a decline in physical and mental health and appearance of chronic non-communicable diseases (CNCD). Healthy behaviors (regular physical exercise along with consumption of fruits and vegetables) have positive effects on the aging process, modulating the speed of functional decline [4], contributing to the prevention or delaying the appearance of CNCD and its complications [5–7], improving quality of life [4] and reducing the risk of premature mortality [8]. However, the proportion of older adults who report healthy behaviors is lower than in other age groups [9].

Several studies in developed countries have reported that age, education, gender, contextual socioeconomic conditions and health status may have an influence on consuming a healthy diet and participating in regular exercise. For example, female gender, marriage, higher level of education, and high household income are positively associated with a healthy diet [10–12], whereas alcohol and tobacco use, oral and gastrointestinal diseases, physical disability and social isolation are negatively associated with a healthy diet [10–12]. Young males with a high educational level have a higher probability of practicing regular physical exercise, whereas a negative association exists among individuals who suffer from a chronic condition and physical exercise [9, 13]. However, such assumptions are controversial. A recent systematic review did not find sufficient evidence to support the association between the determinants previously mentioned and regular physical exercise [13].

In the LAC region, most studies on healthy behaviors were conducted in Brazil and focused on the determinants of healthy behaviors of adults <65 years of age. These studies reported that a growing proportion of adults does not practice physical exercise [14, 15] and does not consume a sufficient amount of fruits and vegetables [16–19]. Factors associated with insufficient physical exercise were female sex, older age, low educational level, high income, smoking, obesity, and self-reported poor health status [14, 15, 20, 21], whereas consumption of fruits and vegetables increased with age, education, and number of household members [17, 18].

From 2006 to 2012, the health status of persons >60 years of age in Mexico worsened. The proportion of overweight and obese older adults increased from 69.2 to 71.7 % [22, 23], and the proportion of physically inactive individuals increased by 9.1 % [15]. During the same period, the prevalence of chronic conditions increased [24]. These complex circumstances justify the search for further evidence to better understand the underlying factors that promote or hamper healthy behaviors of older adults.

Consistent with recommendations from other countries [25, 26], recent dietary and physical activity guidelines from the National Academy of Medicine of México recommend daily consumption of three portions of vegetables and two portions of fruits and at least 150 min of moderate aerobic physical exercise per week for adults ≥65 years of age. However, information on fruit and vegetable consumption and physical exercise in this age group is scarce [27].

The objective of this study was to analyze the factors associated with regular physical exercise and routine consumption of fruits and vegetables, and both healthy behaviors among Mexican older adults. Such information is relevant to elucidate prospective strategies aimed at improving the health status of older adults by addressing the associated modifiable factors.

## Methods

We carried out a secondary data analysis using the baseline data (2014) of the "Cohort of Obesity, Sarcopenia and Frailty of Older Mexican Adults” (COSFOMA) study, which is affiliated with the Mexican Social Security Institute (Instituto Mexicano del Seguro Social, IMSS). The research protocol was approved by the National Committee of Scientific Investigation as well as by the Ethics Committee for Health Investigation (COMBIOETICA09CE101520130424) of the IMSS (No. 2012-785-067). The IMSS is a mandatory social security system for salaried employees in the formal labor market in Mexico and offers a comprehensive package of benefits including health care at all levels (e.g., primary and hospital care) and economic benefits such as a retirement pension. IMSS-insured workers and their first-degree relatives are affiliated with an assigned Family Medicine Clinic (FMC) based on their residence. In 2015, Mexico had 119 million inhabitants; 59 % were affiliated with the IMSS. Furthermore, in Mexico City there were 8.9 million inhabitants; 9.8 % were older adults [28] and half of these older adults were affiliated with the IMSS.

In the COSFOMA study, 1252 adults ≥60 years of age were chosen through a simple random selection from the list of older adults affiliated with the 48 IMSS FMCs in México City. Study participation was carried out using invitation letters sent to older adults at their home addresses to inform them of the nature of the study and to provide them with the survey appointment at their FMC. In those cases in which the older adult did not attend the appointment, a phone invitation was made and, in some cases, a home visit. The response rate was 80.9 % (*n* = 1252) of 1547 older adults were contacted. Data collection was performed by trained healthcare professionals (physician, nurse, nutritionist, psychologist and dentist) through a comprehensive geriatric assessment via a questionnaire and physical examination.

Excluded from the analysis were 301 (24.3 %) older adults who had Mini-Mental Status Examination scores 23 or lower in order to avoid re-call bias of those with cognitive impairment [29]. The final analysis included 948 older adults.

The study had three dependent variables: 1) regular physical exercise defined as at least 3 h per week of any type of physical exercise during leisure time; e.g., brisk walking, jogging, swimming, dancing (physical exercise did not include housework or gardening); 2) routine consumption of fruits and vegetables defined as eating two servings of fruits and three servings of vegetables (or more) daily, and 3) routine performance of both healthy behaviors. Recommendations of the National Academy of Medicine of México served as a reference to evaluate consumption of fruits and vegetables [27].

Questions that included the information about healthy behaviors were as follows:

1. How much time did you routinely spend on leisure time physical activity/exercise during the last year? Response options were as follows: none or <15 min/week, 15–30 min/week, 31–59 min/week, 1–2 h/week, 3–4 h/week, 5–6 h/week and >6 h/week.
2. Please indicate the frequency of consumption of the following foods during the last year such as cabbage, lettuce, spinach, and other vegetables. The complete list of fruits and vegetables available in Mexico and specified as the quantity of one serving was presented. For each type of fruit and vegetable, the response options were as follows: (i) none or less than once/month; (ii) 1–3 times/month; (iii) once/week; (iv) 2–4 times/week; (v) 5–6 times/week; (vi) once/day; (vii) 2–3 times/day; (viii) 4–5 times/day; (ix) >6 times/day.

The conceptual framework of the study was based on the social contextual framework that explains health behavior as complex interrelationships of individual characteristics (biological, psychological, attitudes/beliefs, skills) with social context factors (social ties, social support and neighborhood environment, etc.) [30]. This framework guided the selection of independent variables that included the following:

(i) Participants’ general characteristics such as gender, age group (60–74 and ≥75 years of age), education (elementary school or less and secondary school or higher), employment status and fixed monthly income. The latter variable was defined as all types of regular monthly income identified by older adults and included salary, pensions, and financial support from family members or from the government (e.g., the national program “65+” for persons ≥65 years old).
(ii) Health-related factors comprised the presence of chronic diseases, depression, physical limitations and self-rated health status, as well as a history of regular physical exercise at the age of 50 years. The presence of CNCD was identified through self-report. To facilitate the response, participants were asked if they suffered from any CNCD included in a list of the 15 most frequent CNCD. The revised version of the Center for Epidemiologic Studies–Depression Scale (CES-D) was used to measure depression [31]. This scale contained 35 items and was previously adapted in a sample of older Mexican adults [32]. The CES-D algorithm allows assigning the responses into five groups: (a) without clinically significant symptoms of major depressive episode (MDE), (b) subthreshold MDE symptoms, (c) possible MDE, (d) probable MDE, and (e) clinically relevant symptoms of MDE. The analysis included only older adults with clinically significant symptoms of a major depressive episode. Clinically significant symptoms were identified when the older adult presented dysphoria or anhedonia for at least 2 weeks and any of the following symptoms: significant changes in appetite, sleep disorders, agitation or psychomotor delay, fatigue, excessive or inappropriate guilt and suicidal ideation [32].
  The Short Physical Performance Battery (SPPB) served to measure physical limitations [33]. The SPPB measures lower extremity function using tasks that examine three areas of lower extremity function (static balance, gait speed, and getting in and out of a chair). We classified physical limitations using the total summary score as follows: moderate to severe limitations (0–6 points) and without or mild restrictions (≥7 points). The single question: “How do you perceive your overall health?” assessed self-rated health. Categories of the responses were poor (“poor” or “regular”) and good (“good”, “very good” or “excellent”) self-rated health.
(iii) Social context factors included variables of the social network structure and support and risk of social isolation.

The structure of social networks and social support was identified through the number and type of family members and friends. We used the MOS Social Support Survey questionnaire to assess four types of support: tangible, affectionate, emotional/informational and positive social interaction. To obtain a score for each type of support (subscale), we calculated the average of the scores for each item in the subscale. We then transformed it to a 0–100 scale using the following formula: 100 x (observed score − minimal possible score)/(maximum possible score–minimum possible score) [34]. We also identified participants who received each type of support, none or rarely (average score <4.0) and most of the time (average score 4.0 − 5.0). Older adults at increased risk of social isolation were identified using the Lubben Social Network Scale (LSN-6) based on the cut-off point score <12 [35].

### Statistical analysis

Descriptive statistics were used to analyze the characteristics of older adults and their social support. To compare these characteristics according to the healthy behaviors, we used chi-square test for categorical variables and Student *t*-test for continuous variables.

To obtain the adjusted association between healthy lifestyles and independent study variables we performed multiple Poisson regression analysis with robust variance for each of the dependent variables. The rationale to build the model was to include all relevant independent variables based on the Social Contextual Framework. Poisson regression with robust variance was used as “a better alternative for the analysis of cross-sectional studies with binary outcomes than logistic regression because the prevalence ratio is more interpretable” [36]. The robust variance option was used to control for any possible violation of the Poisson distribution assumption [37] and to correct Type I error, even in the case of the possibility of incorrect model specification [38].

Furthermore, we evaluated the interactions between gender*schooling, gender*regular income, gender*social isolation, gender*smoking and gender*history of regular physical activity. This decision took into account the controversial results of previous studies that the effect of the above-mentioned factors on an outcome (health behaviors) may, in some cases, be gender-dependent. The interactions were evaluated by including a product term for the two factors (e.g., gender*schooling) in the model. However, we did not find any significance for tested interactions.

Stata v.10.0 statistical package (Stata Corp., College Station, TX, USA) was used to perform the analysis; *p* <0.05 was considered statistically significant.

## Results

Table 1 presents the general characteristics of 948 older adults according to their healthy behaviors. There were more women (57.2 %) than men, 15.7 % of the sample was 75+ years old, 29.4 % had elementary school education or less, 40.8 % had paid employment, and 87.6 % had a regular monthly income primarily from pensions (43.9 %) and salaries (40.8 %), whereas other types of income were limited. For example, only 2.4 % of older adults reported receiving government support (data not presented).

**Table 1:** General and health related characteristics of Mexican older adults according to their healthy behaviors

Variables	Total	Routine fruit and vegetables consumption		Regular physical exercise		Both healthy behaviors	
	*n* = 948	Yes *n* = 510 (53.8 %)	No *n* = 438 (46.2 %)	Yes *n* = 405 (42.7 %)	No *n* = 543 (57.3 %)	Yes *n* = 219 (23.1 %)	No *n* = 729 (76.9 %)
	%	%	%	%	%	%	%
General characteristics:							
Female gender	57.2	61.2*	52.5	57.8	56.7	63.0*	55.4
75 years of age and older	15.7	15.7	15.8	14.1	16.9	15.1	15.9
Elementary school or less	29.4	28.0	31.1	25.7*	32.2	26.0	30.5
Job	40.8	39.8	42.0	41.7	40.1	40.2	41.0
Regular monthly income	87.6	90.4*	84.0	86.9	88.0	89.0	87.0
Health related characteristics:							
Chronic disease(s)	34.2	34.3	34.0	32.6	35.4	34.2	34.2
DSM-IV Major Depressive Episode	3.5	3.5	3.4	2.2*	4.4	2.3	3.8
Moderate to severe physical limitations	20.0	21.6	18.3	14.6*	24.1	15.1*	21.5
Excellent or good self-rated health	43.0	44.3	41.6	50.4*	37.6	53.0*	40.1
Lifestyle:							
Smoking	10.3	8.6*	12.3	8.6	11.6	6.8*	11.4
History of regular physical exercise at the age of 50 years	46.8	49.0	44.3	74.1*	26.5	74.0*	38.7

* *p* < 0.05

Health-related variables highlighted that 34.2 % of older adults suffered from one or more CNCD, 3.5 % had a major depressive episode, and 20 % had moderate to severe physical limitations. Concerning self-perception of their health status, 43 % reported excellent or good health, whereas 57 % considered that their health was average or poor.

Regarding health-related behaviors, 10.3 % were current smokers, 53.8 % routinely consumed fruits and vegetables, 42.7 % practiced regular physical exercise, and 46.8 % reported practicing regular physical exercise at the age of 50 years. Only 23.1 % reported both healthy behaviors (routine consumption of fruits and vegetables and regular physical exercise).

Bivariate analysis revealed differences between those with and without healthy behaviors. Women and older adults with a regular monthly income were those who more often reported regular consumption of fruits and vegetables. Adults with a history of physical exercise since the age of 50 years and self-perceived good health practiced physical exercise on a regular basis. On the contrary, those with elementary school education or less and those with moderate to severe physical limitations and depression reported practicing exercise regularly less often. Furthermore, among participants who practiced both healthy behaviors, most were females. They perceived themselves with good health and had a history of regular physical exercise at the age of 50 years.

Table 2 shows the information of the family members of older adults and social support according to their healthy behaviors. Most of these older adults did not live alone. Some reported living with two to four family members (44.9 %). Some had a spouse (57.8 %), children (59.6 %) and friends (71.5 %). Only 14 % of older adults lived alone. A significant proportion of participants reported having a social support network. Emotional/informational support was less frequent (80.4 %), although 33.9 % of older adults were at increased risk of social isolation.

**Table 2:** Family members and social support of Mexican older adults according to their healthy behaviors

	Total	Routine fruit and vegetables consumption		Regular physical exercise		Both healthy behaviors	
	*n* = 948 %	Yes *n* = 510 %	No *n* = 438 %	Yes *n* = 405 %	No *n* = 543 %	Yes *n* = 219 %	No *n* = 729 %
Number of family members living with older adult in the household							
Without family members	14.0	15.1	12.8	14.3	13.8	14.6	13.9
1 family member	27.8	27.1	28.8	29.4	26.7	26.9	28.1
2-4 family members	44.9	45.9	43.6	43.5	45.9	44.3	45.0
5 or more family members	13.3	12.0	14.8	12.8	13.6	14.2	13.0
Type of family members:							
Spouse/life partner	57.8	56.5	59.4	60.2	56.0	60.3	57.1
Children	59.6	60.0	59.1	57.0	61.5	61.2	59.1
Other family members	35.8	35.3	36.3	31.9*	38.7	30.6*	37.3
Friends	71.5	71.8	71.2	77.0*	67.4	78.1*	69.5
Social support (SS):							
Tangible SS: Most of the time	82.9	83.7	82.0	82.2	83.4	82.6	83.0
Affectionate SS: Most of the time	86.8	88.2	85.2	88.1	85.8	89.5	86.0
Positive social interaction: Most of the time	82.0	82.9	80.8	83.0	81.2	83.6	81.5
Emotional/informational SS: Most of the time	80.4	82.0	78.5	80.5	80.3	81.3	80.1
Increased risk of social isolation	33.9	30.8*	37.4	30.1*	36.6	24.7*	36.6

*p < 0.05

The comparison between participants with and without healthy behaviors elucidated that among those who consumed fruits and vegetables on a regular basis, few were at increased risk of social isolation. Among older adults practicing regular physical exercise and those reporting both healthy behaviors, there was a higher prevalence of participants with friends and few were at increased risk of social isolation.

Table 3 shows the results of the multiple Poisson regression (PR) analysis. We found that being female was the factor associated with an increased likelihood of practicing both healthy behaviors when analyzed separately and together (PR 1.19, 95 % CI: 1.04–1.36; PR 1.19, 95 % CI: 1.03–1.37 and PR 1.55, 95 % CI: 1.21–1.97, respectively). Also, good self-rated health was associated with an increased likelihood of regular physical exercise and both healthy behaviors (PR 1.25, 95 % CI: 1.08–1.44 and PR 1.42, 95 % CI: 1.12–1.80, respectively). In addition, regular monthly income (PR 1.37, 95 % CI: 1.10–1.71) was associated with an increased likelihood of routine fruit and vegetable consumption, whereas history of regular physical exercise at the age of 50 years was associated with an increased likelihood of regular physical exercise (PR 3.13, 95 % CI: 2.61–3.76) and both healthy behaviors (PR 3.10, 95 % CI: 2.35–4.08).

**Table 3:** Factors associated with Mexican older adults’ healthy behaviors (Multiple Poisson regression with robust variance) (*n* = 948)

	Routine fruit and vegetables consumption			Regular physical exercise			Both healthy behaviors		
	Adjusted PR	95 % CI	*p*	Adjusted PR	95 % CI	*p*	Adjusted PR	95 % CI	*p*
Individual factors:									
Female gender	**1.19**	**1.04–1.36**	**0.009**	**1.19**	**1.03–1.37**	**0.016**	**1.55**	**1.21–1.97**	**0.000**
75 years of age and older	0.97	0.82–1.14	0.693	1.00	0.82–1.23	0.996	1.04	0.76–1.43	0.809
Elementary school or less	0.95	0.83–1.10	0.515	0.96	0.82–1.13	0.613	0.97	0.74–1.26	0.799
Job	0.99	0.88–1.12	0.909	0.99	0.87–1.13	0.903	0.98	0.78–1.24	0.902
Regular monthly income	**1.37**	**1.10–1.71**	**0.005**	0.92	0.77–1.11	0.385	1.15	0.82–1.63	0.418
Presence of chronic disease(s)	1.02	0.90–1.16	0.697	0.99	0.86–1.14	0.894	1.09	0.87–1.38	0.447
DSM-IV Major Depressive Episode	1.10	0.80–1.51	0.565	0.79	0.49–1.27	0.337	0.88	0.44–1.79	0.736
Moderate to severe physical limitations	1.12	0.97–1.29	0.113	0.84	0.68–1.04	0.111	0.87	0.62–1.20	0.396
Good self-rated health	1.04	0.92–1.17	0.554	**1.25**	**1.08–1.44**	**0.002**	**1.42**	**1.12–1.80**	**0.004**
Smoking	0.84	0.67–1.05	0.131	0.87	0. 68–1.10	0.250	0.72	0.45–1.13	0.149
History of regular physical exercise at the age of 50 years	1.11	0.98–1.24	0.090	**3.13**	**2.61–3.76**	**0.000**	**3.10**	**2.35–4.08**	**0.000**
Social context factors:									
Number of family members living with older adult in the household:									
Without family members	1.45	0.92–2.27	0.108	0.78	0.47–1.28	0.329	0.91	0.40–2.06	0.821
1–2 family members	1.22	0.87–1.70	0.246	0.82	0.56–1.20	0.244	0.73	0.40–1.34	0.317
3–4 family members	1.17	0.94–1.46	0.168	0.89	0.69–1.15	0.371	0.77	0.52–1.15	0.208
5 or more family members	Ref.			Ref.					
Type of family members:									
Spouse/life partner	1.07	0.91–1.27	0.415	1.10	0.91–1.33	0.307	1.28	0.94–1.76	0.120
Children	1.13	0.91–1.41	0.259	0.87	0.68–1.10	0.256	1.16	0.76–1.77	0.479
Other family members	1.07	0.90–1.28	0.410	0.86	0.70–1.06	0.162	0.78	0.56–1.09	0.140
Friends	0.92	0.80–1.07	0.283	1.13	0.94–1.36	0.190	1.05	0.78–1.42	0.739
Social support (SS):									
Tangible SS: Most of the time	0.96	0.77–1.20	0.706	0.88	0.72–1.09	0.248	0.84	0.59–1.18	0.309
Affectionate SS: Most of the time	1.09	0.84–1.43	0.506	1.06	0.80–1.41	0.675	1.16	0.73–1.86	0.530
Positive social interaction: Most of the time	0.94	0.74–1.20	0.633	1.04	0.83–1.30	0.709	1.01	0.67–1.52	0.975
Emotional/informational SS: Most of the time	1.07	0.85–1.35	0.555	0.95	0.77–1.16	0.602	0.93	0.65–1.31	0.669
Increased risk of social isolation	0.87	0.75–1.01	0.074	1.03	0.88–1.22	0.689	0.77	0.57–1.03	0.077

*PR* prevalence ratio. The bold values highlight the statistically significant multivariable-adjusted prevalence ratios

## Discussion

Our main findings indicate that 53.8 % of older Mexican adults affiliated with the IMSS reported routine consumption of fruits and vegetables and 42.7 % participated in regular exercise, although only 23.1 % reported engaging in both healthy behaviors. Women and older adults with a fixed income and those adults with a self-perception of good health and history of regular physical exercise at the age of 50 years had an increased likelihood of practicing both healthy behaviors.

We found that a low percentage of older Mexican adults practiced physical exercise regularly and consumed the recommended amount of fruits and vegetables. A recent systematic review reported that the worldwide prevalence of adequate physical activity among older adults ranges from 2.3 to 83 % [9]. Information from Latin America is scarce; however, important variations exist depending on the location and age of the sample of older adults. For example, in Chile, between 28 % (Valparaíso) and 35 % (Chillan City) of older adults were physically active [39]. In Brazil, the prevalence ranged from 23.7 % (70 years of age or older) to 42.7 % (60–69 years of age) [14]. In Colombia, the prevalence was 62.4 % [40]. Regarding dietary patterns, only 20 % of older Brazilian adults reported routine consumption of fruits and vegetables [16, 18]. Information on regular physical exercise and fruit and vegetable consumption from other Latin American countries is absent.

Older Mexican females had an increased probability of practicing both healthy behaviors. Studies in developed countries have reported that women of different ages eat more fruits and vegetables than men [41–43] but practice less physical exercise [13]. However, the explanation for this fact remains controversial [41, 42]. Our results indicate that older Mexican women eat more fruits and vegetables and had a high probability of practicing regular physical exercise, which is congruent with few studies that reported similar results [44, 45]. We observed that men had a lower consumption of fruits and vegetables and practiced less physical exercise than women. This finding should prompt effective approaches to promote healthy behaviors in older Mexican males.

This recommendation is congruent with several other studies reporting that men engaged more frequently in unhealthy behaviors, delayed seeking medical help when ill [46] and had a lower life expectancy compared to women [47]. Furthermore, a recent review on strategies for engaging men in chronic disease prevention and management programs [48] showed that traditional group-based programs focused on topics such as nutrition and physical activity were often seen by men as inherently feminine, thus representing a barrier for participation. Some facilitators to gain men’s participation are history of negative health event, personal concern for health status, and motivation to improve physical appearance. Other factors to engage men were a group component with like-minded men, use of humor in the delivery of health information, inclusion of both nutrition and physical activity components, and the presence of some type of competition [48]. However, the above-mentioned review did not include studies from Latin America and from the population of older men; therefore, research in Mexico and Latin America aimed at investigating older men’s beliefs, attitudes and facilitators towards healthy behaviors is important. Also, it seems reasonable to promote the implementation of a multiple health behavior change intervention aimed at improving eating practices and physical activity simultaneously to maximize its potential impact [49].

In our study, receiving a fixed monthly income was a significant factor related to the routine consumption of fruits and vegetables. This finding is consistent with prior studies that reported the importance of regular income in order to purchase healthy foods [50]. Promoting consumption of healthy foods would be feasible in older adults affiliated with the IMSS because most of these adults have a fixed income from pensions and salaries. However, implementation of educational programs to modify dietary habits should take into account other determinants such as availability of food, cultural and individual preferences, and affordability of competitive basic needs.

Healthy aging requires adherence to healthy eating and regular exercise [51]. The relationship between healthy aging and healthy behaviors is bi-directional. Congruent with previous studies [9, 11, 12], we found that good self-rated health was associated with both healthy behaviors. Another finding that supports this statement was the history of regular physical exercise at the age of 50 years that was associated with current regular exercise and both healthy behaviors. Therefore, it is crucial to promote healthy behaviors at early ages so that, as the population ages, older adults can live healthier lives and attain and preserve good health.

Mexico is a country with a collectivist culture. This may help to explain that, in our study, a high proportion of older adults (86 %) reported living with family members, having friends (71.5 %) and having a social support system (from 80.4 % for emotional support to 86.8 % for affectionate support). Nonetheless, in our study we found that the characteristics of structure and support of the social networks of older adults were not associated with their healthy behaviors. This finding is consistent with a previous literature review on the determinants of physical exercise in older adults that found insufficient evidence for an association between baseline social support and regular physical exercise [13].

This study has both strengths and limitations. The inclusion of a variety of factors in the analysis, particularly multiple variables to describe the social network structure and social support, represents a strength of the analytical approach.

The study has several limitations worth mentioning. First, self-reporting served to evaluate both healthy behaviors, which could lead to misclassification. However, it would be expected that any misclassification would be non-differential and would have biased our results toward the null hypothesis, thus underestimating the strength of associations between the evaluated factors and both healthy behaviors. It was not practical to perform an objective measure of physical activity (e.g., accelerometers) and short-term-recall instruments (e.g., diaries or previous day recalls). It has been recognized that such methods can introduce systematic and random measurement errors. The use of objective devices is also associated with high costs and activity monitoring. Also, short-term recall instruments require a high cognitive demand and participant burden of obtaining a sufficient number of repeated measures to estimate common daily activity levels [52]. Therefore, such methods represent a difficult application in older adults and in limited-resource settings.

In addition, this study is a secondary data analysis, which reduces the possibility of an in-depth exploration of other factors included in the conceptual model that can be associated with healthy behaviors of older adults. Particularly, we did not obtain in-depth information about participants’ socioeconomic status or characteristics of their residential areas that could be included in the analysis to ascertain its association with healthy behaviors of older adults [30].

Third, the study design was cross-sectional; thus, it was not possible to make inferences about causal relationships or the direction of the association between the factors and healthy behaviors.

Fourth, the physical activity evaluation did not include occupational, housework or gardening activities and included only physical exercise. This fact may help to explain why men in our study have lower levels of physical activity than women, despite the fact that other studies suggest otherwise.

Finally, the generalizability of the study is limited to older adults (and their first-degree relatives) affiliated with the IMSS and who are active or retired employees in the formal labor sector. This population does not include those who work in the informal labor sector, those who are unemployed and persons living in rural areas because they are affiliated with the governmental “Seguro Popular” health insurance program providing healthcare benefits for the uninsured population. Although the study does not have national representativeness, IMSS affiliates represent 60 % of the country’s population. IMSS members’ income is variable, ranging from 1 to 25 times the minimum wage. In 2011, the average salary of an IMSS affiliate was 4.3 minimum wages; only 8.5 % of affiliates had an income of 10 or more minimum wages [53]. Therefore, it may be expected that our findings can be applied to the high percentage of the Mexican population with different socioeconomic levels as well as the populations with similar characteristics, e.g., older adults affiliated with the contributory scheme of the Comprehensive Social Security System in Colombia.

## Conclusions

In regard to the consumption of fruits and vegetables along with the habitual practice of physical activity, we may conclude that a small percentage of older adults affiliated with the IMSS engage in these healthy behaviors as recommended, specifically due to the fact that most of these subjects have a regular income and a social network. The significant proportion of older adults who do not routinely consume fruits and vegetables and do not participate in regular physical exercise calls for effective approaches to promote healthy behaviors. It is relevant to conduct further research-based interventions that take into account the contextual factors to promote healthy behaviors. Prevention programs should also emphasize the importance and benefits of healthy behaviors from early ages.

## Abbreviations

CES-D: Center for epidemiologic studies–depression scale
CNCD: Chronic non-communicable diseases
COSFOMA: Cohort of obesity, sarcopenia and frailty of older mexican adults
FMC: Family medicine clinic
IMSS: Mexican Institute of Social Security
LAC: Latin America and Caribbean
LSN-6: Lubben social network scale
MDE: Major depressive episode
SPPB: Short physical performance battery
